# CMTM3 Overexpression Predicts Poor Survival and Promotes Proliferation and Migration in Pancreatic Cancer

**DOI:** 10.7150/jca.57082

**Published:** 2021-08-03

**Authors:** Zixuan Zhou, Zuyi Ma, Zhenchong Li, Hongkai Zhuang, Chunsheng Liu, Yuanfeng Gong, Shanzhou Huang, Chuanzhao Zhang, Baohua Hou

**Affiliations:** 1 South China University of Technology School of Medicine, Guangzhou 510006,Guangdong Province, China.; 2 Department of General Surgery, Guangdong Provincial People's Hospital, Guangdong Academy of Medical Sciences, Guangzhou 510080, China.; 3 Shantou University of Medical College, Shantou 515000, China.; 4 The Second School of Clinical Medicine, Southern Medical University, Guangzhou 510515, China.

**Keywords:** CMTM3, pancreatic cancer, prognosis, cancer aggressiveness

## Abstract

**Background:** Recent evidence has shown that CKLF-like MARVEL transmembrane domain containing 3 (CMTM3) promoted carcinogenesis and tumor progression in a variety of cancer types. The goal of our study is to investigate the association between CMTM3 and pancreatic cancer (PC).

**Materials and Methods:** In current study, data from public databases was used to analyze CMTM3 expression in PC. Quantitative real-time polymerase chain reaction (qRT-PCR) and immunohistochemistry (IHC) were used to investigate CMTM3 expression and determine its clinical significance in PC. Then CMTM3 promoting PC aggressiveness was demonstrated *in vitro* experiments by cell proliferation and migration assay. Functional and pathway enrichment analyses were performed to evaluate the potential role of CMTM3 in PC.

**Results:** Results of qRT-PCR and IHC revealed that CMTM3 was significantly overexpressed in PC tissues. High CMTM3 expression was an independent risk factor for poor prognosis of PC patients. Overexpression of CMTM3 was associated with poor overall survival (P-value =0.031) and disease-free survival (P-value =0.0047) in the TCGA cohort. Functional and pathway enrichment analyses showed that CMTM3 were enriched in “Regulation of cell proliferation and regulation of cell differentiation, cell morphogenesis, regulation of cell differentiation, Hedgehog signaling pathway, Wnt signaling pathway, ECM-receptor interaction and pathways in cancer”. In PC cell lines, CCK8, clone formation and transwell assays showed that CMTM3 knockdown inhibited cells proliferation and migration.

**Conclusion:** CMTM3 was overexpressed and promotes tumor aggressiveness in PC. Our findings provided a novel therapeutic target for PC.

## Introduction

Pancreatic cancer (PC) is a malignant tumor and the fourth most common cause-related mortality worldwide, with an incidence rate equaling that of its mortality 1, 2. We are facing a difficult scenario in treating pancreatic cancer: the increasing incidence and the poor prognosis with 5-year survival rate lower than 3% 3. Although novel target therapy like poly ADP-ribose polymerase (PARP) inhibitor was discovered, there were still limited knowledge for tumor behavior and specific biomarkers at the molecular level 4. Therefore, better understanding of molecular mechanisms that involve in regulating PC development and progression will help to find predictive biomarkers and improve survival for PC patients 5.

CKLF-like MARVEL transmembrane domain-containing family (CMTM) consists of eight members, some of which were shown to be dysregulated in human cancer 6. Previous studies found CMTM proteins were involved in critical biological processes in cancer development, including growth factor receptor activation and recycling, cell proliferation, and immune evasion 7. In particular, CMTM3 is one of the chemokine-like factor genes located in a cluster on chromosome 16q22, which exhibits critical functions in the immune system, male reproductive system and tumorigenesis 8. It has recently been shown that CMTM3 was silenced or down-regulated in gastric, breast, and renal carcinomas 9-11. However, the expression of CMTM3 in pancreatic cancer is unknown and the association between CMTM3 expression and the prognosis and clinicopathological features of PC patients remains unclear.

In the current study, we explored the expression of CMTM3 and its role in predicting patients' survival for PC. Further, bioinformatic analysis and *in vitro* experiments were conducted to investigate the effect of CMTM3 on tumor cell behavior and the potential mechanism.

## Material and Methods

### Datasets and data acquisition

The gene expression data recorded based on Fragments Per Kilobase per Million (FPKM) and clinical information for 178 PC samples were obtained from the Cancer Genome Atlas (TCGA, https://portal.gdc.cancer.gov/repository) database up to September 2020. GSE62165 dataset based on GPL9115 (including 13 pancreas and 118 PC samples), GSE15471 dataset based on GPL13667 (including 36 pancreas and 36 PC samples), and GSE62452 dataset based on GPL6244 (including 69 pancreas and 69 PC samples) were downloaded from Gene Expression Omnibus (GEO, https://www.ncbi.nlm.nih.gov/geo/) database for validation. The expression level of CMTM3 in PC was further analyzed using the Oncomine database (https://www.oncomine.org/resource/main.html) and the Gene Expression Profiling Interactive Analysis (GEPIA, https://gepia.cancerpku.cn/index.html).

### PC Samples collection and tissues microarray construction

PC tissues and normal adjacent tissues (NATs) were collected from Guangdong Provincial People's Hospital, Guangdong Academy of Medical Sciences (GPPH cohort) from 2014 to 2019. The inclusion criteria were as follows: (1) all the patients underwent R0 pancreaticoduodenectomy (1 mm without cancer); (2) no neoadjuvant treatment was performed; (3) clinicopathological information and a followed-up visit were available; (4) all the tumor tissues were pathologically confirmed as PC. Our study was approved by the Ethics Association of Guangdong Provincial People's Hospital, and signed informed consent was obtained from each patient before participation in the research. Each sample was evaluated by professional pathologists. These samples were stored at -80 °C until required.

### Functional and pathway enrichment analysis

GeneMANIA tool (http://genemania.org/) was used to analyze the relationship of CMTM3 with its neighbor genes and construct a network map at the gene level. STRING (https://string-db.org/) and co-expressed genes screened from cBioPortal database were integrated to DAVID 6.7 (https://david-d.ncifcrf.gov/) to perform Gene Ontology (GO) analysis and Kyoto Encyclopedia of Genes and Genomes (KEGG) pathway analysis. Results were visualized by using R software (version 3.5.3) with “ggplot2” package and a P-value <0.05 was considered statistically significant.

### Cox regression and survival analysis

Multivariate Cox proportional hazards regression was used to evaluate independent prognostic factors of PC prognosis in GPPH cohort. log-rank tests and Kaplan-Meier analyses were performed using the survival R package between the high and low expression group in both TCGA cohort and GPPH cohort to assess the predictive ability for patients' survival. Overall survival (OS) was defined as the period between surgical resection to death or the last contact. Disease-free survival (DFS) was defined as the period between the resection to any form of tumor recurrence or metastasis 9-11. The median follow-up time of patients from GPPH cohort was 25.5 months (range, 16-78 months).

### RNA extraction and quantitative real-time polymerase chain reaction (qRT-PCR)

Total RNA was isolated from tissues and cells using the Qiagen RNeasy Mini Kit in combination with oncolumn DNase treatment (Applied Biosystems, USA). A High Capacity RNA-to-cDNA Kit (Applied Biosystems) was used to synthesize the first strand of cDNA. Quantitative real-time PCR was performed using the Power SYBR Green PCR Master Mix (Applied Biosystems) with gene-specific primers. According to the manufacturer's instructions, the total RNA was extracted with TRIzol reagent (USA, NY, USA). qRT-PCR was performed using the SYBR Green.

Detection RT-PCR System (TaKaRa, Japan) was used to assess the following CMTM3 primers: forward primer, GCTTGTGCTGGCCCATGATG-3. reverse primer, TGTGGGCTGTGGTCTCATCT. GAPDH was used as the reference control and was amplified with the following primers: forward primer, GGTGTGAACCATGAGAAGTATGA; reverse primer, GAGTCCTTCCACGATACCAAAG. The relative mRNA expression level was determined by the 2^-∆∆Ct^ method. All qRT-PCR experiments were conducted in triplicate.

### Western blot

The cells were washed twice with 4 °C PBS and then lysed in cold RIPA buffer with protease inhibitors. Protein concentrations were determined using the BCA Protein Assay Kit (Pierce, Rockford, IL, USA). The total protein was transferred to a nitrocellulose membrane after denaturing by 10% SDS-PAGE. The membranes were blocked with 5% nonfat milk in Tris-buffered saline containing 0.1% Tween-20 (TBST) for 1 h at room temperature. The membranes were then incubated with the primary antibodies overnight at 4 °C. The membranes were washed three times with TBST and then incubated with secondary antibodies (anti-rabbit IgG) for 1 h at room temperature. The membranes were washed three times with TBST, and then, the targeted proteins were detected by the ECL reagent (EMD Millipore, MA, USA) method.

### Cell cultural and infection

Human pancreatic cancer cell lines (SW1990, AsPC-1, PANC-1, BxPC-3 and Capan-2) were purchased from Procell (https://www.procell.com.cn/, China, Wuhan), and the immortal human pancreatic duct epithelial cell line (HPDE6) was a gift from South China University of Technology School of Medicine. Cells were cultured in RPMI 1640 medium (Gibco) with 10% fetal bovine serum, at 37 °C and CO_2_.

### Cell transfection

The CMTM3 knockdown vector was constructed by Shanghai Genechem (Shanghai, China). For packaging of the construct, 293T cells were transfected with GV112 by Helper1.0 Packaging Plasmid Mix, and after 3 days, the virus particles were collected with Lenti-Concentin Virus. Precipitation Solution was conducted according to the packaging protocol of SBI. Cells were infected with Trans virus transduction reagent. The 293T knockout plasmid and nontargeting control plasmid were constructed with the following target sequences: GV112-NC-1 CCGGTTCTCCGAACGTGTCACGTTTCAAGAGAACGTGACACGTTCGGAGAATTTTTG. The human CMTM3 cDNA was cloned into hU6-MCS-CMV-puro lentiviral vector. Positive cells were identified by puromycin screening. The infection efficiency was determined by counting the number of GFP-positive cells which should be guaranteed to be > 90%.

### Immunohistochemistry (IHC) assays and evaluation

Paraffin-embedded PC tissues were consecutively sectioned at 4-μm intervals and then mounted on polylysine-coated glass slides. The slides were subsequently incubated for 2 hours at 62 °C, deparaffinized, and rehydrated. Heat-mediated antigen retrieval was performed in 10 mM Tris-citrate buffer (pH 7.0) in a pressure cooker. Endogenous peroxidase activity was blocked by incubating the sections with 3% hydrogen peroxide for 10 minutes at room temperature. Each section was then incubated with 5% normal goat serum in phosphate buffered saline containing 0.1% Tween 20 for 1 hour at room temperature to block nonspecific binding of the primary antibody. The slides were subsequently incubated with primary antibodies (diluted 1:50) against CMTM3 (NBP2-68944) overnight at 4°C. After washing, each slide was incubated with the appropriate horseradish peroxidase-labeled secondary antibody and then developed with 3,3′-diaminobenzidine solution (GeneTech, Shanghai, China) before counterstaining with hematoxylin. CMTM3 is localized in the nucleus and cytoplasm. Staining intensity was scored as 0, 1, 2, or 3 for absent, weak, moderate, or strong, respectively. The staining percentage was given a score of 0 (absent) for less than 5% positive staining, 1 (focal) for 5% to less than 25% positive staining, 2 (diffuse) for at least 25% to less than 50% positive staining, or 3 (diffuse) for at least 50% positive staining. The sum of the intensity and distribution scores was then used to determine CMTM3 immunoreactivity. A score of 1 or 0 was considered to show low expression, whereas higher scores were considered to indicate high expression. Two pathologists independently assessed the specimens. Images were obtained using an Olympus BX63 microscope (Olympus, Tokyo, Japan). The immunohistochemical score (H score) was calculated by multiplying the positive cell score by the staining intensity score.

### Cell proliferation assay

Cell counting Kit-8 assay (CCK8) was used to quantify cell proliferation following manufacturer's instructions. Briefly, 1,500 cells / 96-well plates were seeded. Next day, each well was added with the CCK-8 solution [2-(2-methoxy-4-nitrophenyl)-3-(4-nitrophenyl)-5-(2,4-disulfophenyl)-2H-tetrazolium, monosodium salt] which forms a formazan dye upon reduction in the presence of an electron mediator. After 4 h at 37 °C in 5% CO2 incubator, the absorbance (OD 450 nm) was assessed in a microplate reader (BioRad). Data represents the mean ± SD from three independent experiments.

### Transwell migration assay

Transwell chambers (Bd Biosciences, San Jose, CA, USA) were used for the analysis of cell migration. A total of 5 ×10^4^ cells in 200 ul serum-free DMEM were seeded on the upper chambers and dMEM with 10% FBS was added to the lower chamber. After 24 h of incubation, the invaded cells in the lower side of the membranes were fixed with methanol and stained with crystal violet (Beyotime). Images were acquired using an inverted microscope. Invaded cells were counted from three different fields. Data represents the mean ± SD from three independent experiments.

### Clone formation assay

For colony formation assays, 500 cells of each type were seeded into six well culture plates, gently shaken and incubated at 37 °C in a 5% CO2 incubator for 10 days. Subsequently, the medium was removed and the cells were stained with 0.1% crystal violet (Sigma, St. Louis, MO) to quantify positive colonies (diameter >40 µm) after imaging. The differences in colony formation ability of different cell types were documented in triplicate.

### Data analysis

The significance of continuous parameters presented as the mean ± SD was determined by Student's t-test. χ^2^ test or Fisher's exact test were used to explore qualitative variables as appropriate. All statistical analyses were performed using R software Version 4.0.1 (https://www.r-project.org/) and SPSS software Version 24.0 (SPSS, Inc., Chicago, IL, USA). A P-value <0.05 was considered statistically significant.

## Results

### Overexpression of CMTM3 in pancreatic cancer

**Figure 1:**
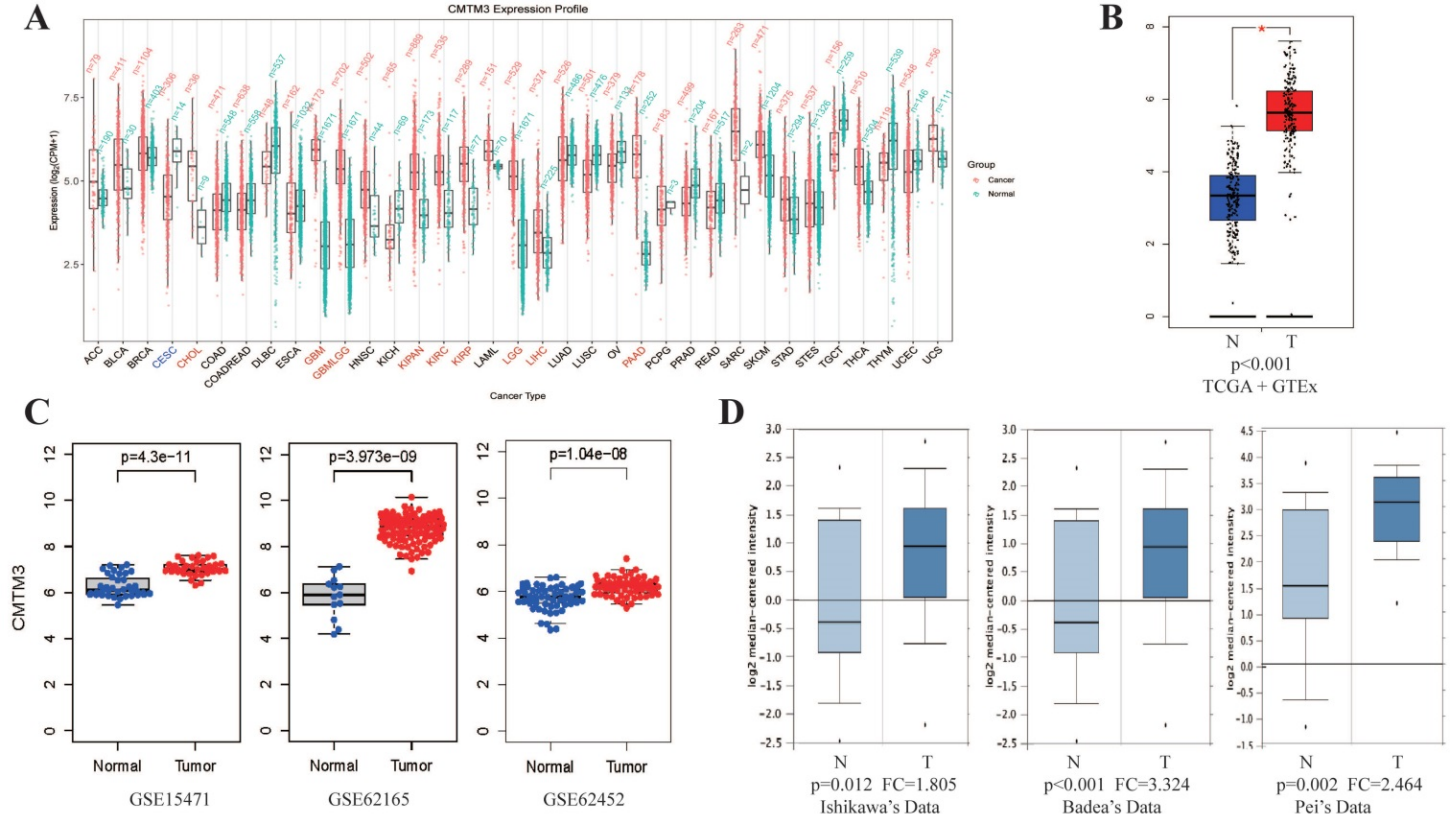
** Expression of CMTM3 in public database. (A)** The analysis of CMTM3 differential expression in several cancer types in TCGA+GTEX database. **(B)** The over-expression (red) and down-regulated (blue) of CMTM3 in pancreatic cancer (PC) in TCGA+GTEX database. **(C)** The analysis of CMTM3 differential expression in PC in three GEO datasets. **(D)** The analysis of CMTM3 differential expression in PC in three Oncomine datasets. T: Tumor; N: Normal.

By analyzing CMTM3 expression in PAN-cancer database of GEPIA, we found CMTM3 was overexpressed in most cancer types especially in PC (**Figure 1A-B**). Similar results was also found in three GEO datasets (GSE15471, GSE62165 and GSE62452) and in 3 individual datasets of the Oncomine (Ishikawa's Dataset, Badea's Dataset and Pei's Dataset) (**Figure 1C-D**).

**Figure 2:**
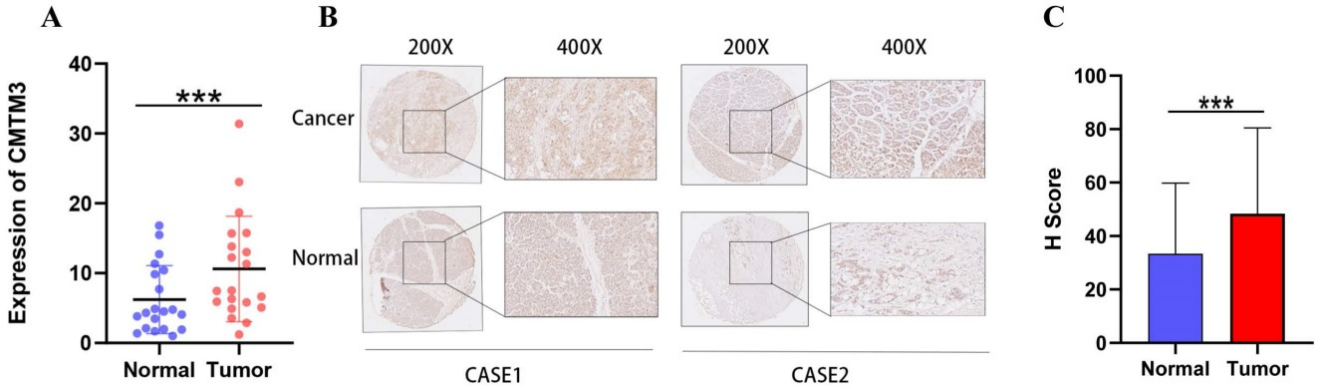
**Upregulation of CMTM3 in pancreatic cancer (PC) tissue. (A)** The mRNA expression levels of CMTM3 in PC tissues and normal tissues of 20 samples. **(B)** Representative images of CMTM3 staining in PC specimens and normal tissues. **(C)** H score of CMTM3 staining in PC specimens and normal tissues.

We next performed Real-time PCR and IHC to investigate the mRNA and protein level of CMTM3 for the human PC samples in our institute. The results showed that CMTM3 mRNA was significantly upregulated in PC tissues compared to normal pancreas tissues (**Figure 2A**). The immunohistochemistry (IHC) results and H scores confirmed that higher expression of CMTM3 in PC tissues (**Figure 2B-C**).

### CMTM3 correlates with unfavorable clinical characteristics and predicts poor survival for PC

**Figure 3:**
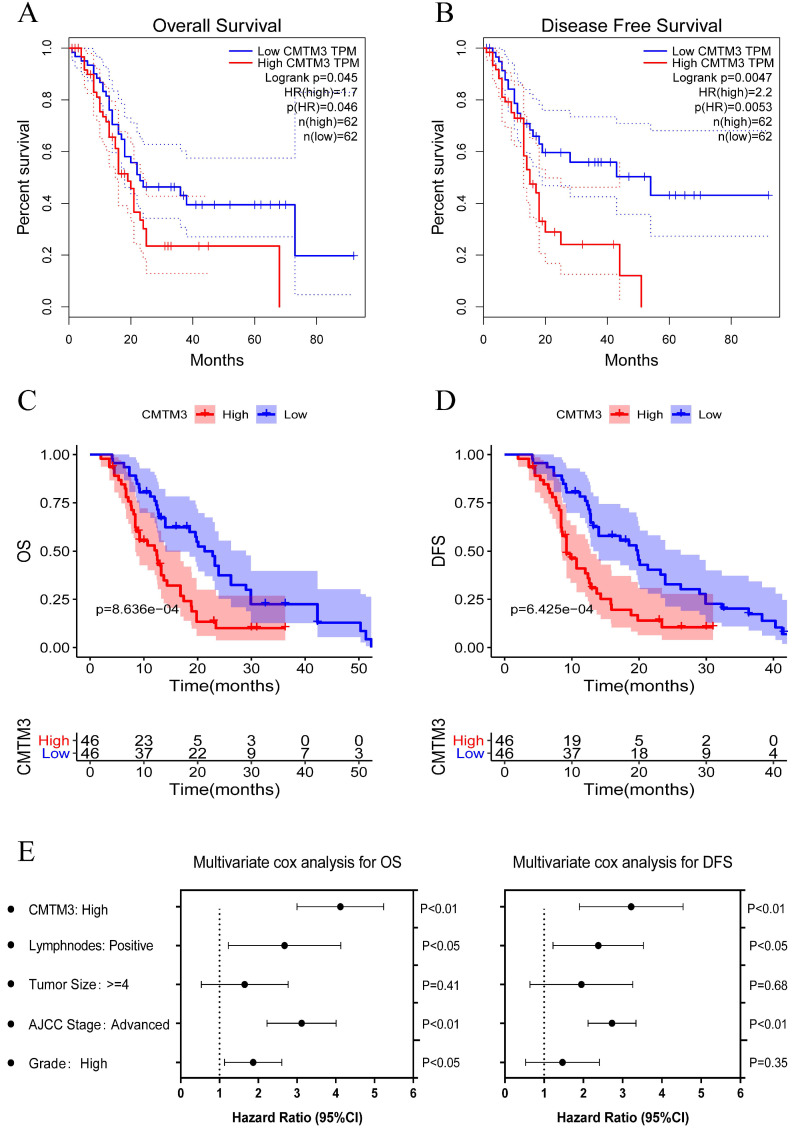
** Kaplan-Meier analyses for overall survival (OS) and disease-free survival (DFS) were performed between the high and low CMTM3 expression groups with a median cut-off. (A-B)** The high expression group was associated with poorer OS (p=0.031) and DFS (p=0.0047) in the TCGA cohort. **(C-D)** The high expression group was associated with poorer OS (p=8.636e-04) and DFS (p=6.425e-04) in the GPPH cohort. **(E)** Multivariate cox regression analysis showed CMTM3 overexpression was the independent prognostic indicator for OS and DFS in patients with PC.

To further explore the role of CMTM3 in PC progression, we investigated the association between CMTM3 expression and patients' clinical characteristics in TCGA cohort and GPPH cohort (**Table 1**). It's indicated that high expression of CMTM3 was correlated with low pathological grade and high recurrence/metastasis rate. To determine the prognostic value of CMTM3, PC cohorts were divided into high and low expression groups with a median cut-off and Kaplan-Meier analyses were performed between the groups. The results showed the high expression group was associated with poorer OS (P-value =0.031) and DFS (P-value =0.0047) in the TCGA cohort (**Figure 3A-B**). The similar results were found in GPPH cohort (**Figure 3C-D**). Further, univariate and multivariate cox regression analyses were performed and the results showed that CMTM3 overexpression was the independent prognostic indicator for OS and DFS in patients with PC (**Figure 3E and Tables 2 & 3**). Briefly, the data in Table 1 and Figure 3 indicated CMTM3 overexpression correlates with unfavorable clinical characteristics and predicts poor survival for PC.

### Neighbor gene network and functional enrichment analyses of CMTM3

**Figure 4:**
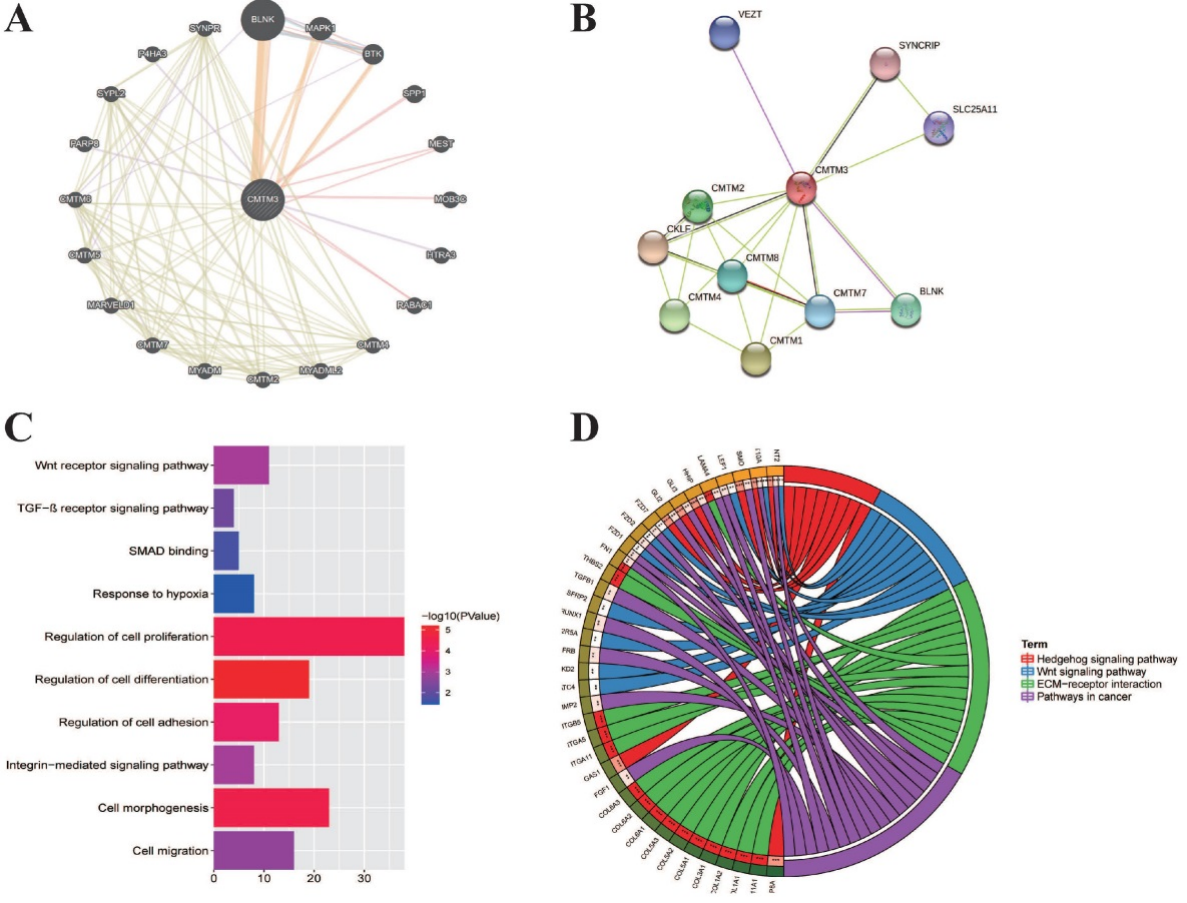
** The interaction network and functional enrichment analysis of CMTM3 in pancreatic cancer. (A)** Neighbor gene network of CMTM3. (B) Protein-protein interaction (PPI) network of CMTM3. (C) Gene Ontology (GO) enrichment analysis of CMTM3. (D) Kyoto Encyclopedia of Genes and Genomes (KEGG) pathway analysis of CMTM3.

We next explore the molecular function of CMTM3 in PC. The neighbor genes of CMTM3 presented as a network map were shown by GeneMANIA tools (**Figure 4A**). In detail, the top 20 genes which have correlation with CMTM3 included CMTM1-8, BLNK, MKPK1, BTK, SPP1, MEST, MOB3C, HTRA3, RABAC1, MYADML2, MYADM, MARVELD1, PARP8, SYPL2, P4HA3, and SYNPR. Using STRING tools, we analyzed the relationship of CMTM3 family members and constructed a network map at the protein level (**Figure 4B**). We found CMTM3 was connected with CMTM1, CMTM2, CMTM3, CMTM4, CMTM7, and CMTM8.

In addition, we explored the function of CMTM3 by analyzing its potential biological pathways in PC. The co-expression analyses for CMTM3 were performed by using cBioPortal dataset (Spearman's correlated coefficient >0.5 or <-0.5, P-value <0.05) and 457 co-expression genes for CMTM3 were enrolled into DAVID 6.7 and subjected to functional and pathway enrichment analyses. GO enrichment analysis showed that CMTM3 may be involved in “Wnt receptor signaling pathway, TGF-β receptor signaling pathway, SMAD binding, Response to hypoxia, Regulation of cell proliferation, differentiation and adhesion, Integrin-mediated signaling pathway, cell morphogenesis and regulation of cell differentiation” (**Figure 4C**). In KEGG analysis, CMTM3 was found to be mainly enriched in “Hedgehog signaling pathway, Wnt signaling pathway, ECM-receptor interaction and pathways in cancer” (**Figure 4D**).

### CMTM3 promotes proliferation and migration of PC cells *in vitro*

**Figure 5:**
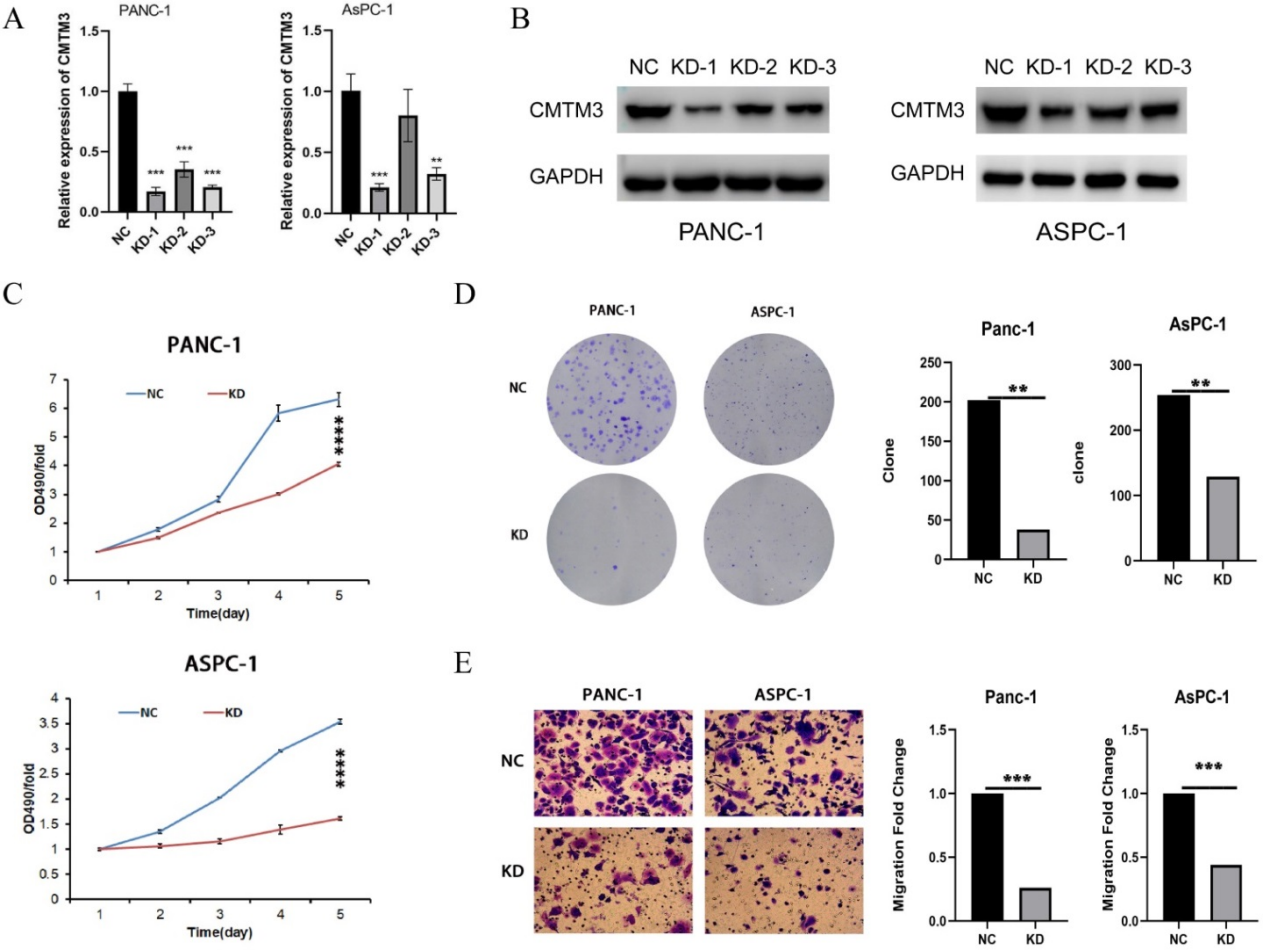
** Inhibition of CMTM3 repressed the proliferation and migration of pancreatic cancer cells.** (A-B) RNA extraction and quantitative real-time polymerase chain reaction (A) and western blot (B) were used to detect the down-regulation of CMTM3 in PANC-1 and AsPC-1 cells knockdowned by three short interfering RNAs. CCK-8 assay (C) and Colony formation assay (D) showed that CMTM3 down-regulation reduced the proliferation in Panc-1 and AsPC-1 cells. (E) Transwell assay showed knockdown of CMTM3 reduced the cell migration of PC cells in Panc-1 and AsPC-1 cells.

To experimentally validate the function of CMTM3 in PC, we constructed three shRNAs targeting the back-splice site of CMTM3 to specifically downregulate the expression of CMTM3 in Panc-1 and AsPC-1 cells (**Figure 5A-B**). Based on the knock down efficiency, we used the shRNA-1 subclones for further cell experiments. We found knockdown of CMTM3 significantly inhibited cell proliferation compared to the NC subclones in both Panc-1 and Aspc-1 cells, as indicated by the CCK-8 assay and colony formation assay (Panc-1, P-value <0.001; AsPC-1, P-value <0.001) (**Figure 5C and 5D**). Moreover, transwell assays showed knockdown of CMTM3 reduced the cell migration of PC cells in Panc-1 and AsPC-1 cells (P-value <0.001; AsPC-1, P-value <0.001) (**Figure 5E**). Taken together, these findings suggested that CMTM3 is vital to proliferation and migration of PC cells.

## Discussion

Finding novel effective biomarkers or molecule regulating PC initiation and progression is of great value for discovering new therapeutic target 13, 14. CMTM family (CMTM1-8) has been reported to be differentially expressed between tumor and normal tissue, thus suggesting that CMTMs may actively regulate tumor development in various cancer types 15-17. The functions of CMTM family proteins in tumor growth, metastasis, and antitumor immunity are well recognized 15-17. In addition, CMTM family proteins play crucial roles in mediating the clinical characteristics of tumors, including promoting chemotherapeutic resistance in non-small cell lung cancer (NSCLC), and have prognostic value in multiple cancers 21, 22. CMTM3, which is a member of the CMTM family, was first identified by Han et al. in 2003. CMTM3 is associated with the pathogenesis of multiple carcinomas 23. Previous studies indicated that CMTM3 was silenced or down-regulated in gastric, breast, nasopharyngeal, esophageal, colon and renal carcinomas and its expression inversely correlates with grade and phase in prostate cancer when combined with IL30 study 23-25. These studies found that restoration of CMTM3 inhibits the cancer cells that above-mentioned proliferation and migration, invasion *in vitro* and tumor growth *in vivo* 26. However, the expression of CMTM3 in PC and its association with prognosis of PC remains unknown 27.

In the current study, we observed that CMTM3 was proportionally overexpression in pancreatic cancer tissues and identified the effects of CMTM3 restoration on PC cells proliferation and migration and invasion *in vitro*. And CMTM3 overexpression was associated with low pathological grade, high recurrence/metastasis rate and worse patients' survival, indicating a prognostic value of CMTM3 in PC. GO enrichment analysis showed that CMTM3 may be involved in “Regulation of cell proliferation and regulation of cell differentiation, cell morphogenesis and regulation of cell differentiation”. Furthermore, CMTM3 was found to be closely related to “Hedgehog signaling pathway, Wnt signaling pathway, ECM-receptor interaction and pathways in cancer” 28, 29. The hedgehog signaling is a stem cell-related pathway that plays a crucial role in embryonic development, tissue regeneration, and organogenesis. Aberrant activation of hedgehog signaling leads to pathological consequences, including a variety of human tumors such as pancreatic cancer 28, 29. Multiple lines of evidence indicate that blockade of this pathway with several small-molecule inhibitors can inhibit the development of pancreatic neoplasm 30. In addition, activated hedgehog signaling has been reported to be involved in fibrogenesis in many tissues, including the pancreas 31. We suppose that CMTM3 and hedgehog signaling with the specific inhibitor cyclopamine may have highlight new insights on their potential relationship with respect to the development of novel targeted therapies. On the other hand, tumors with inactive Wnt signaling are a heterogeneous group displaying interaction of chromosomal instability, Wnt signaling, and epigenetics 29. In this study, we supposed that CMTM3 may be a gene of Wnt signaling pathways and associated to some pathways in cancer progression along with CCND3, PPP3CA, and PPP3CC.

There are several limitations of our study. For example, although the expression of CMTM3 was identified as prognostic biomarkers for DFS and OS in the study, further prospective experiments and *in vivo* studies are need to validate our results and explore underlying molecular mechanisms.

To sum up, we demonstrated that increased CMTM3 expression is an unfavorable marker in PC survival. CMTM3 regulated proliferation, migration, apoptosis. In-depth mechanistic studies suggested that CMTM3 have regulation with cell proliferation. Collectively, our present study provides novel insights into the mechanism of tumorigenesis in PC, as well as a vital biomarker for diagnosis and a potential target for the treatment of PC.

## Figures and Tables

**Table 1:** Correlation between CMTM3 and clinicopathological features in pancreatic cancer

Characteristics	CMTM3 expression					
	TCGA cohort			GPPH cohort		
	High (n=83)	Low (n=83)	P value	High (n=46)	Low (n=46)	P value
**Age**			0.6324			0.8169
<60	34	30		12	14	
≥60	49	53		34	32	
**Gender**			0.7556			0.5312
Male	43	46		22	26	
Female	40	37		24	20	
**AJCC stage**			0.4717			0.2023
I	7	12		12	6	
II	71	66		31	34	
III	1	2		2	4	
IV	3	1		0	2	
Unkonwn	1	2		1	0	
**Histologic grade**			**0.01866**			**0.008396**
G1	8	21		3	5	
G2	50	45		28	39	
G3	24	14		12	2	
G4	1	1		0	0	
Unkonwn	0	2		3	0	
**Tumor size**			0.4611			0.09651
<4	49	53		8	16	
≥4	29	23		38	30	
Unkonwn	5	7		0	0	
**Lymph nodes positive**			0.1605			0.7093
Yes	63	54		16	14	
No	18	27		28	32	
Unkonwn	2	2		2	0	
**Tumor site**			0.07104			**0.04429**
Head of Pancreas	70	58		26	16	
Body/Tail of Pancreas	9	18		18	29	
Unkonwn	4	7		2	1	
**Recurrence/Metastasis**			**0.02629**			**0.009009**
Yes	28	15		18	6	
No	46	60		28	40	
Unkonwn	9	8		0	0	
**Diabete mellitus**			0.1551			0.7664
Yes	21	13		15	13	
No	50	59		30	33	
Unkonwn	12	11		1	0	
**Alcohol history**			0.6226			0.169
Yes	47	46		25	18	
No	28	34		19	27	
Unkonwn	8	3		2	1	

**Table 2:** Univariate and multivariate Cox regression analysis of risk factors associated with overall survival

Clinicopathological variables	Univariate analysis			Multivariate analysis		
	HR	95% CI	P Value	HR	95% CI	P Value
CMTM3 expression (High vs. Low)	6.02	4.39-7.65	**<0.01**	4.09	3.02-5.16	**<0.01**
Gender (Male vs. Female)	1.72	0.68-2.76	0.32			
Age (≥50 vs. <50)	1.27	0.51-2.03	0.59			
Tumor site (Head vs. Body/Tail)	1.31	0.91-1.71	0.09			
Tumor size (≥4 cm vs. <4 cm)	1.53	1.17-1.89	**<0.05**	1.64	0.50-2.78	0.41
Lymphnodes positive (Yes vs. No)	3.21	1.98-4.44	**<0.01**	2.66	1.27-4.06	**<0.05**
AJCC stage (Advanced vs. Early)	3.47	2.16-4.78	**<0.01**	3.20	2.38-4.02	**<0.01**
Histologic grade (High vs. Low)	2.12	1.20-3.04	**<0.05**	1.89	1.12-2.66	**<0.05**

**Table 3:** Univariate and multivariate Cox regression analysis of risk factors associated with disease-free survival

Clinicopathological variables	Univariate analysis			Multivariate analysis		
	HR	95% CI	P Value	HR	95% CI	P Value
CMTM3 expression (High vs. Low)	5.61	3.91-7.01	**<0.01**	3.24	1.92-4.56	**<0.01**
Gender (Male vs. Female)	1.31	0.43-2.19	0.72			
Age (≥50 vs. <50)	1.42	0.78-2.06	0.31			
Tumor site (Head vs. Body/Tail)	1.56	0.89-2.23	0.13			
Tumor size (≥4 cm vs. <4 cm)	1.89	1.21-2.57	**<0.05**	2.00	0.68-3.32	0.68
Lymphnodes positive (Yes vs. No)	2.96	1.86-4.06	**<0.01**	2.47	1.26-3.68	**<0.05**
AJCC stage (Advanced vs. Early)	3.21	2.09-4.33	**<0.01**	2.72	2.09-3.35	**<0.01**
Histologic grade (High vs. Low)	2.03	1.17-2.89	**<0.05**	1.47	0.47-2.47	0.35

## Abbreviations

PC: Pancreatic Cancer
PC: Pancreatic Ductal Adenocarcinoma
CMTM3: CKLF-like MARVEL transmembrane domain containing 3
DEGs: Differentially Expressed Genes
GEPIA: Gene Expression Profiling Interactive Analysis
TCGA: the Cancer Genome Atlas
GTEx: the Genotype-Tissue Expression
FPKM: Fragments Per Kilobase per Million
GO: Gene Ontology
KEGG: Kyoto Encyclopedia of Genes and Genomes
TILs: Tumor-Infiltrating Lymphocytes
MHC: Major Histocompatibility Complex
